# Downregulation of pectin biosynthesis gene *GAUT4* leads to reduced ferulate and lignin-carbohydrate cross-linking in switchgrass

**DOI:** 10.1038/s42003-018-0265-6

**Published:** 2019-01-17

**Authors:** Mi Li, Chang Geun Yoo, Yunqiao Pu, Ajaya K. Biswal, Allison K. Tolbert, Debra Mohnen, Arthur J. Ragauskas

**Affiliations:** 1 BioEnergy Science Center, Oak Ridge National Laboratory (ORNL), 1 Bethel Valley Road, Oak Ridge, TN 37831 USA; 2 Joint Institute for Biological Sciences, Biosciences Division, ORNL, 1 Bethel Valley Road, Oak Ridge, TN 37831 USA; 3 Center for Bioenergy Innovation, ORNL, 1 Bethel Valley Road, Oak Ridge, TN 37831 USA; 4 Department of Biochemistry and Molecular Biology and Complex Carbohydrate Research Center, University of Georgia, 315 Riverbend Road, Athens, GA 30602 USA; 5 School of Chemistry and Biochemistry and Renewable Bioproducts Institute, Georgia Institute of Technology, 500 10th Street NW, Atlanta, GA 30332 USA; 6 Department of Chemical and Biomolecular Engineering, University of Tennessee, 1512 Middle Drive, Knoxville, TN 37996 USA; 7 Department of Forestry, Wildlife, and Fisheries, Center for Renewable Carbon, University of Tennessee Institute of Agriculture, 2506 Jacob Drive, Knoxville, TN 37996 USA; 8 Present Address: Department of Paper and Bioprocess Engineering, State University of New York College of Environmental Science and Forestry, Syracuse, NY 13210-2781 USA

**Keywords:** Biofuels, Agriculture

## Abstract

Knockdown (KD) expression of *GAlactUronosylTransferase 4* (*GAUT4*) in switchgrass improves sugar yield and ethanol production from the biomass. The reduced recalcitrance of *GAUT4*-KD transgenic biomass is associated with reduced cell wall pectic homogalacturonan and rhamnogalacturonan II content and cross-linking, and the associated increases in accessibility of cellulose to enzymatic deconstruction. To further probe the molecular basis for the reduced recalcitrance of *GAUT4*-KD biomass, potential recalcitrance-related factors including the physicochemical properties of lignin and hemicellulose are investigated. We show that the transgenic switchgrass have a lower abundance of ferulate and lignin-carbohydrate complex cross-linkages, reduced amounts of residual arabinan and xylan in lignin-enriched fractions after enzymatic hydrolysis, and greater coalescence and migration of lignin after hydrothermal pretreatment in comparison to the wild-type switchgrass control. The results reveal the roles of both decreased lignin-polymer and pectin cross-links in the reduction of recalcitrance in *PvGAUT4*-KD switchgrass.

## Introduction

Lignocellulosic biomass is superior to fossil-fuel based carbon as a resource for production of bioenergy, bioproducts, and biomaterials in biobased economies because its use results in less environmental damage and improved greenhouse gases reduction^1^. Transition from fossil-based to lignocellulose-based fuels has been challenged by the inherent recalcitrance of biomass—the natural resistance of plant cell walls to microbial and enzymatic deconstruction to fermentable sugars or products^2^. A pretreatment step is usually necessary to disrupt the plant cell wall matrix and increase the accessibility of the structural polysaccharides to enzymes^3^. While the development of pretreatment technologies has been advanced in the past decades in an attempt to achieve desirable process economics for bioenergy, another strategy via plant genetic manipulation has also been successfully used to design engineered plants with reduced biomass recalcitrance^4^. The ultimate aim of developing such engineered plants is to make ideal feedstock for bioenergy so that the need for pretreatment can be reduced or eliminated, which could notably lead the way towards achieving economically viable lignocellulosic liquid fuels production.

The *GAlactUronosylTransferase 4* (*GAUT4*) gene belongs to the *GAUT1*-related gene family, which encodes enzymes responsible for the biosynthesis of pectic polysaccharides and pectic glycans in more complex cell wall matrix polymers^5,6^. Pectin, a family of complex polysaccharides containing α-d-galacturonic acid, contributes to the structural integrity and strength of plant cell walls. The effect of *GAUT1-*related genes on the cell wall compositional phenotypes has been revealed from the altered wall polysaccharides pectin and xylan in the *GAUT* mutants^7^. A reduction of *GAUT4* activity in tomato resulted in taller plants with higher vegetative biomass, with the suppression of this gene possibly resulting in a looser cell wall^8^. Down-regulation of the *GAUT12* gene in *Populus* led to reduced pectin and xylan contents, increased biomass yield, and more importantly, enhanced sugar release^9^. Recently, the down-regulation of *GAUT4* genes in switchgrass was shown to result in plants with reduced recalcitrance including 14–15% higher total sugar release and 41–65% higher ethanol yield compared with the controls, along with substantially enhanced growth^10^. The results indicated that the *GAUT4* gene is associated with switchgrass cell wall recalcitrance and that the increased plant growth of the *GAUT4*-knockdown (*GAUT4*-KD) lines is accompanied by reduced cell wall integrity, loosened cell walls, reductions in cell wall pectic homogalacturonan, and rhamnogalacturonan II, as well as reductions in the affected inter- and intra-polymer cross-linking^10^.

Lignification is generally conceived of as an inward process starting from the middle lamella and primary cell wall which usually contains large amounts of pectin^11,12^. Earlier studies have revealed that pectin is distributed and somehow covalently attached with lignin throughout wood cell walls^13^. In addition, the irregular distribution of pectin in the middle lamella corresponds to the pattern of lignin deposition in alfalfa cell walls^14^. It is well known that the relatively intractable lignin plays an important role in contributing to biomass recalcitrance by encapsulating polysaccharides in cell walls^15,16^. By removing and/or redistributing lignin during biomass pretreatment, the polysaccharide fractions in the cell wall can become more accessible and amenable to enzymatic hydrolysis. Structural alterations of lignin can impact cell wall recalcitrance^16^. For instance, a study of 47 natural *Populus* variants found that sugar release depended on lignin content and S/G ratio, with sugar release yields generally increasing with increasing S/G ratio and decreasing lignin content^17^. An earlier study found that the suppressed expression of *GAUT12* in *Populus* resulted in lignin content comparable to controls but with increased S/G ratio, a factor that may be associated with the reduced recalcitrance in the *GAUT12*-KD transgenic lines^9^. Another lignin and hemicellulose-related factor, lignin-carbohydrate complex (LCC) cross-linkages, has also been proposed to correlate with biomass recalcitrance^16,18^. For example, the reduction in ferulate (FA)-mediated cross-linking of lignin-polysaccharides in maize^19^ and silage^20^ improved digestibility. Other studies have also demonstrated that LCC linkages are closely associated with biomass recalcitrance^21,22^. There is evidence suggesting that pectin could cross-link to hemicellulose (xylan/xyloglucan) and phenolics (FA/*p*-coumarate (*p*CA))^23,24^. However, the potential effect of pectin biosynthesis on lignin and hemicellulose structure in *GAUT*-silenced plants has never been examined.

We therefore have investigated structural changes in lignin, as well as hemicellulose characteristics, in *GAUT4*-KD engineered switchgrass that demonstrated reduced recalcitrance^10^. To understand the relationship between the down-regulation of *GAUT4* and its recalcitrance phenotype in regards to lignin and hemicellulose structure, we have used gel permeation chromatography (GPC) and nuclear magnetic resonance (NMR) spectroscopy to characterize and compare the structures of cell wall constituents of three *GAUT4*-KD transgenic lines (2A, 2B, and 4A) and one wild-type control plant. We found that the *GAUT4*-KD lines have a lower abundance of FA and LCC cross-linkages (phenyl glycoside), reduced amounts of residual arabinan and xylan in lignin-enriched fractions after enzymatic hydrolysis, reduced hemicellulose molecular weight, and greater coalescence and migration of lignin after hydrothermal pretreatment in comparison to the wild-type switchgrass control. The results of these analyses identify novel effects of down-regulation of *GAUT4*-catalyzed pectin biosynthesis that have implications regarding the mechanisms of reduced biomass recalcitrance and the complexities of cell wall formation.

## Results

### Hemicellulose characteristics of *GAUT4*-KD switchgrass

**Fig. 1:**
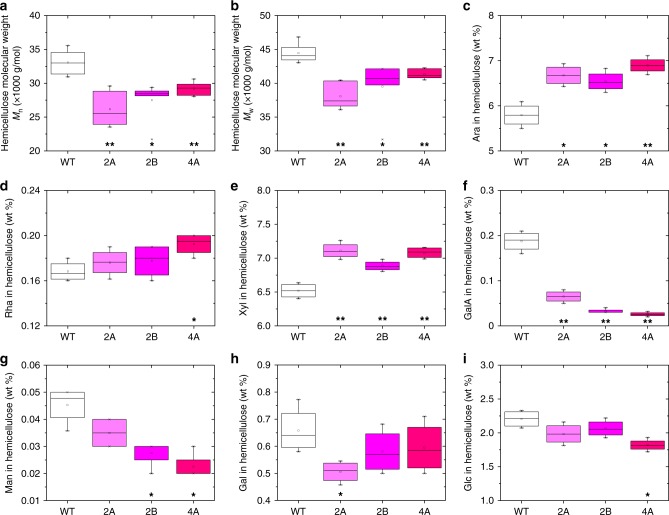
The molecular weights and glycosyl residue composition of the hemicellulose fractions from switchgrass. Number-average (*M*_n_) and weight-average (*M*_w_) molecular weights of the alkaline-extracted hemicellulose-enriched fractions isolated from *GAUT4-*KD (2A, 2B, and 4A) lines and wild-type (WT) control. Ara: arabinose; Rha: rhamnose; Xyl: xylose; GalA: galacturonic acid; Man: mannose; Gal: galactose; Glc: glucose. *p value <0.05 and **p value <0.001 by Student’s *t* test of biologically duplicate values

Cross-linkages between polysaccharides and phenolics may have a role in plant cell wall structural and functional complexity associated with biomass recalcitrance. The pectic polysaccharides have been reported to be cross-linked with hemicellulose, for example, xylan in soybean and xyloglucan in Arabidopsis^23^. We tested the hypothesis that KD of the *GAUT4* pectin biosynthesis gene in switchgrass may influence the structure of hemicellulose by comparing the molecular weight of hemicellulose extracted from transgenic lines and control. We found that the molecular weights of the hemicellulose-enriched fractions extracted by alkaline from the *GAUT4-*KD lines were lower than those in comparable fractions from the control plants (Fig. 1a and 1b). The number-average (*M*_n_) and weight-average (*M*_w_) molecular weight of the hemicellulose-enriched fractions ranged from 25,000–35,000 to 35,000–45,000 g mol^−1^, respectively, with the *M*_n_ and *M*_w_ of hemicellulose-enriched polymers from the transgenic lines being 12–20% and 7–14% smaller, respectively, compared with the wild-type control. Similarly, Li et al.^25^ reported that the reduced recalcitrance of *caffeic acid O-methyltransferase* down-regulated switchgrass had a 7–11% reduction in hemicellulose molecular weight compared with the control plants^25^. According to previous reports^26,27^, two-dimensional heteronuclear single quantum coherence (2D HSQC) NMR analysis showed that the hemicellulose of switchgrass is primarily composed of α-d-glucurono-l-arabino-d-xylans. The HSQC (Supplementary Fig. 1) and ^13^C NMR spectra (Supplementary Fig. 2) of the hemicellulose-enriched fractions from the *GAUT4-*KD lines are almost identical to the wild-type control. However, quantitative analysis of the hemicellulose fraction revealed that the *GAUT4-*KD lines had higher Ara and Xyl and lower GalA residual contents compared with the wild-type control (Fig. 1). The result is consistent with our previous paper in light of increased Ara and reduced GalA in the transgenic lines versus the wild-type control^10^. The results suggest that down-regulation of *GAUT4* activity and reduced expression of *GAUT4* genes in switchgrass had an effect not only on the sugar compositions but also on the molecular weights of hemicellulose-enriched fractions. The reduction of *M*_n_ and *M*_w_ of hemicellulose is possibly associated with the reduction of homogalacturonan and rhamnogalacturonan II and their inter-polymer cross-linking with xylan, leading to smaller molecular size. In addition, hemicellulose with lower *M*_n_ and *M*_w_ values may have shorter chains and more reducing ends, potentially making it more prone to exo-xylanase action^28^. Furthermore, lower molecular weight hemicellulose may have reduced hydrogen bonding to cellulose or other wall polymers, thereby possibly leading to a less cross-linked wall. Thus, a higher efficiency of sugar release could be expected upon the hydrolysis of such transgenic lines with hemicellulose-containing deconstruction enzyme mixtures^10^. It is therefore possible that the reduction of hemicellulose molecular weight in the *GAUT4* switchgrass contributes to the reduced cell wall recalcitrance.

### Lignin characteristics of *GAUT4*-KD switchgrass

**Fig. 2:**
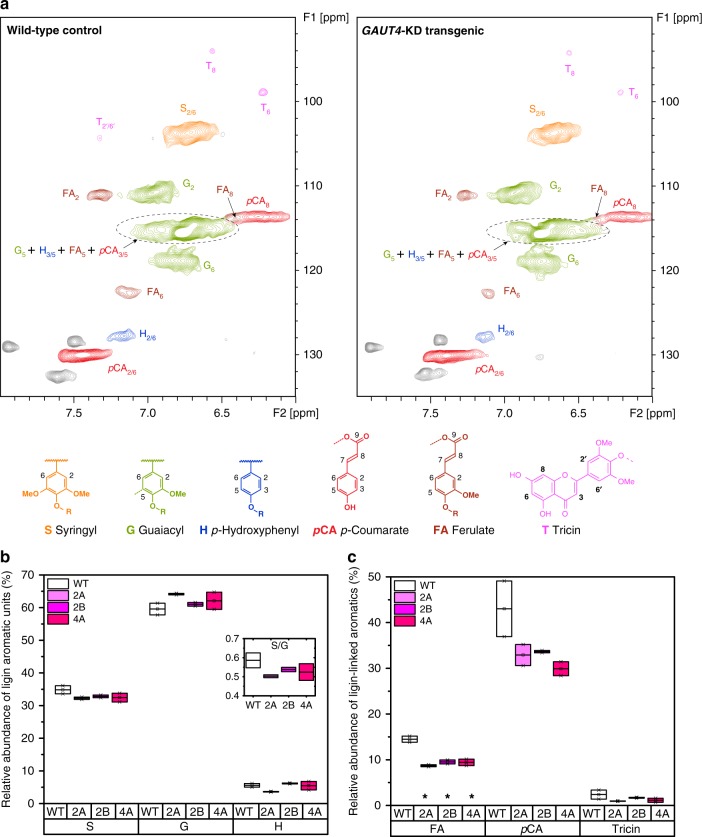
Relative abundance of lignin aromatic units in the *GAUT4*-KD and control switchgrass. **a** Aromatic regions from 2D HSQC NMR spectra of lignin isolated from wild-type (WT) control and *GAUT4-*KD (2A, 2B, and 4A). **b** Relative abundance of lignin subunits (insert is lignin S/G ratio). **c** Relative abundance of lignin hydroxycinnamates and lignan (tricin) from 2D HSQC NMR spectra. *p value <0.05 by Student’s *t* -test of biologically duplicate values

Although there was minimal difference in the total amount of lignin (wt%) in the *GAUT4*-KD lines compared with the control switchgrass^10^, lignin structure is also an important factor that can contribute to biomass recalcitrance^16^. Cellulolytic enzyme lignin (CEL) has been widely used to represent the intact lignin properties in previous studies^29–31^. We thus analyzed the CEL monolignol composition to understand the chemical features of lignin in the *GAUT4*-KD lines. The CEL isolated represented 20–24 wt% of the Klason lignin contained in the switchgrass (Supplementary Fig. 3). The glycosyl residue analyses revealed that there were 8–10 wt% neutral sugars in the isolated CEL (Supplementary Fig. 4). The relative abundance of the different lignin subunits (syringyl (S), guaiacyl (G), and *p*-hydroxyphenyl (H), the lignin-associated hydroxycinnamates (*p*CA and FA), and tricin (T), as well as the major inter-unit linkages (β-aryl ether (β-*O*-4), resinol (β-β), and phenylcoumaran (β-5)) were measured by 2D ^13^C-^1^H HSQC NMR spectroscopic analysis (Fig. 2a and Supplementary Fig. 5). The aromatic regions of the HSQC spectra of the switchgrass lignin indicated abundant S, G, and H subunits along with considerable amounts of *p*CA, FA, and T units (Fig. 2a). The ^13^C-^1^H correlation for S_2/6_, G_2_, and H_2_ were observed at δ_C_/δ_H_ 103.4/6.68, 110.7/6.95, and 127.5/7.18 ppm, respectively. As shown in Fig. 2b, *GAUT4* down-regulated switchgrass lignin had slightly lower S/G ratio (0.50–0.54) than the wild-type switchgrass lignin (0.59); however, the difference was not statistically significant. The lignin S/G ratio is an important indicator for biomass recalcitrance^17^, but the S/G ratio and the biomass recalcitrance can have varied correlations depending on the species^15^. For instance, a negative correlation between lignin S/G ratio and recalcitrance was reported with *Populus*^17^. In contrast to *Populus*, Fu et al.^32^ reported a positive correlation of S/G ratio in lignin to recalcitrance of switchgrass genetically modified in *caffeic acid O-methyltransferase* expression. In this study, significant change was not observed in the lignin S/G ratio in *GAUT4* down-regulated greenhouse grown switchgrass compared with the control (Fig. 2b).

The relative abundance of hydroxycinnamates (*p*CA and FA) and tricin in the *GAUT4*-KD switchgrass was also analyzed as *p*CA_2/6_, FA_2_, and T_2′/6′_ (Fig. 2). *p*CA is involved in lignification as a *p*-coumarate-monolignol through the formation of ester bonds at γ-OH^33,34^, and FA forms covalent linkages between polysaccharides and lignin in herbaceous plants^35^. These hydroxycinnamates are important aromatic units of lignin, especially in grasses, since they are correlated to delignification and biomass digestibility^33,34,36^. The relative abundance of *p*CA and FA in the *GAUT4*-KD switchgrass (29.9–33.6% and 8.7–9.5%, respectively) was lower than those in the wild-type control (38.5% and 14.4%, respectively) (Fig. 2c). In particular, FA is generally associated with C5 cross-linkages to arabinosyl residues in arabinoxylans. Therefore, the decreased FA content might have indicated less LCC cross-linkages, possibly leading to lower recalcitrance in the transgenic plants. Besides the lignin subunits and hydroxycinnamates, tricin was also observed in the aromatic regions. Tricin was recently reported to link to lignin by β-*O*-4 linkages and contribute to lignin composition^37,38^. The content of tricin in the transgenic switchgrass (1.0–1.7%) was similar to wild-type (1.2%).

The suppression of *GAUT4* genes in switchgrass also resulted in a slight variation in the lignin inter-unit linkages. The major lignin inter-unit linkages, including β-aryl ether (β-*O*-4), resinol (β-β), and phenylcoumaran (β-5), in the aliphatic regions of the NMR spectra were semi-quantified by integrating the α-position of each unit. As Table 1 presents, β-*O*-4 content over the total aromatic subunits (Ar) was reduced in the *GAUT4*-KD switchgrass, while the contents of C-C linkages (β-5 and β-β) in the transgenic switchgrass were not substantially changed.

**Table 1:** Relative abundance of lignin inter-unit linkages and LCC linkages using ^13^C-^1^H HSQC NMR analysis

Structures	WT	2A	2B	4A
	Ar%	Ar%	Ar%	Ar%
Inter-unit linkages				
β-aryl ether (β-*O*-4)	47.8 (3.8)	42.1 (1.3)	38.0 (3.0)	36.9 (2.4)
Phenylcoumaran (β-5)	7.7 (0.7)	6.4 (0.2)	6.0 (0.5)	6.0 (1.1)
Resinols (β-β)	0.5 (0.0)	0.5 (0.2)	0.8 (0.2)	0.8 (0.1)
LCC				
Phenyl glycoside	3.4 (0.1)	2.1 (0.2)*	2.4 (0.1)*	0.9 (0.1)**
γ-esters	12.3 (2.2)	11.0 (2.0)	6.3 (0.0)*	9.9 (0.8)

*WT* wild-type, *2A, 2B, and 4A* transgenic switchgrass lines, *Ar%* relative content over 100 aromatic subunits (S + G + H), *(value)* standard deviation

* *p* value <0.05

** *p* value <0.001 by Student’s *t* test of biologically duplicate values

LCC cross-linkages have been considered as a contributor to biomass recalcitrance^16,18^. LCCs impede chemical and/or biological deconstruction of plant cell walls; therefore, reduction and/or cleavage of LCC bonds can improve the digestibility of biomass. Benzyl ether LCCs, ester LCCs, and phenyl glycoside LCC linkages were reported in previous studies^39,40^. In this study, CH_2_-γ in γ-esters (63.2/4.21 ppm) and carbohydrates C_1_ associated with phenyl glycoside linkages (103–100/5.20–4.87 ppm) were observed, while the signals of CH-α in benzyl ether (α-ester) structures at 81–80/4.7–4.5 or 81–80/5.1–4.9 ppm were not detected. The content of phenyl glycoside LCC linkages was substantially lower in the transgenic switchgrass plants (Table 1). The γ-ester LCC content in the *GAUT4*-KD was not statistically significantly different from that of the control, except for 2B. Since the signals of LCC γ-esters can be overlapped with the signals of FA and coumarate derivatives in non-wood lignins^39^, the change of γ-ester LCCs in *GAUT4*-KD lines is difficult to quantify at this point. Grass lignin is usually cross-linked to cell walls through hydroxycinnamates (primarily FA) to hemicellulose^41,42^. The signals of the anomeric carbon of arabinan^43^ and xylan^44^ units in each lignin-enriched sample were observed and used to estimate the residual carbohydrates after enzymatic hydrolysis. Consistent with the reduced FA content in *GAUT4*-KD lines, the *GAUT4*-KD switchgrass had less anomeric carbon in arabinan and xylan to total aromatics ratios in the lignin-enriched residues after enzymatic hydrolysis (Supplementary Fig. 6), suggesting reduced LCC linkages and reduced recalcitrance. This result agrees with the glycosyl residues analysis revealing that the CEL of *GAUT4*-KD lines had significantly reduced Ara and Xyl content compared with the wild-type control (Supplementary Fig. 4, Student’s *t* test, *p* < 0.001). Recently, information from cell wall glycan-directed monoclonal antibodies suggested that the disruption of lignin-arabinogalactan/pectin/xylan associations have important roles in reducing biomass recalcitrance^21^. In another study of 12 transgenic hybrid poplars, the strong linear relationship between LCC amount and sugar recovery further demonstrated LCC linkages are remarkably associated with biomass recalcitrance^22^. The reduced amount of LCC linkages implies reduced lignin-polysaccharides cross-linking in the cell wall of the *GAUT4*-KD switchgrass, which is beneficial for lignin removal or redistribution as evidenced by our scanning electron microscopy (SEM) results.

**Fig. 3:**
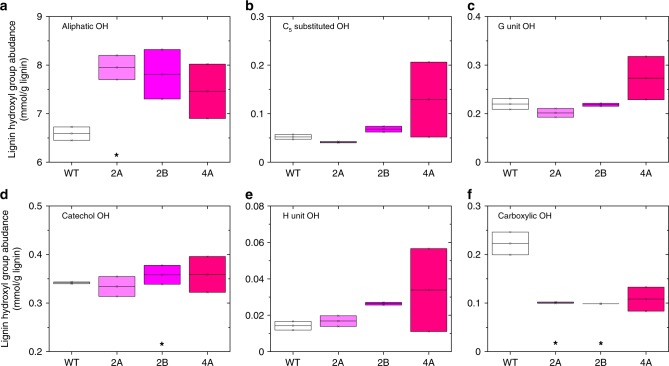
Lignin hydroxyl groups analyzed using ^31^P NMR after phosphitylation. Lignin is isolated from wild-type (WT) control and *GAUT4-*KD (2A, 2B, and 4A). *p -value <0.05 by Student’s *t* test of biologically duplicate values

For a better understanding of the structural features of lignin from the *GAUT4*-KD switchgrass and the wild-type control, these lignin-enriched residues were further purified to CEL that is suitable for ^31^P NMR and molecular weights analyses. ^31^P NMR spectroscopy is an effective tool for differentiating and quantitating the different types of hydroxyl groups (OHs) including aliphatic, carboxylic, guaiacyl, syringyl, C_5_-substituted phenolic hydroxyls, and *p*-hydroxyphenyls in lignin^45^. OHs in lignin are associated with lignin properties such as hydrophobicity and influence the interaction between lignin and enzymes^16^. The content of these various OH was determined and quantified with respect to the internal standard (Fig. 3 and Supplementary Fig. 7). The aliphatic OH dominated the total OH content (ca. 88–91%) in both the wild-type control and *GAUT4*-KD lines. Among the free phenolic OHs, the *p*-hydroxyphenyl was predominant (5% of total free OHs) followed by guaiacyl and C_5_-substituted OH. It should be noted that the high content of *p*-hydroxyphenyl could also be attributed to the OHs from *p*-coumarate and tricin units in switchgrass lignin^46^. A trace amount of catechol OH peaking at 138.9 ppm was observed in switchgrass lignin (Supplementary Fig. 7). Quantitative comparison of OHs between the wild-type control and *GAUT4*-KD switchgrass revealed a significant difference in the content of carboxylic OH (Fig. 3, Student’s *t* test, *p* < 0.05). The transgenic switchgrass had only half of the carboxylic OH content of the wild-type, which are mostly from conjugated carboxylic OH according to chemical shifts study of lignin-related model compounds^47^. The wild-type lignin contained predominantly unconjugated carboxylic OH at ~134.7 ppm. The lower amount of carboxylic OH groups could be related to the reduced FA and *p*CA in the lignin of *GAUT4*-KD lines as evidenced in the aforementioned 2D HSQC NMR results (Fig. 2). The transgenic lines also had higher aliphatic OH content with 7.0–7.8 mmol g^−1^ (6.4 mmol g^−1^ in wild-type control), although the only difference between line 2A and the wild-type showed statistical significance. Other OHs such as *p*-hydroxyphenyl, C_5_ substituted, and catechol OH in the *GAUT4*-KD switchgrass were not significantly different from the values in the wild-type control.

The molecular weights of lignin in the *GAUT4*-KD switchgrass and the wild-type control were measured using GPC analysis. All the isolated lignins from the *GAUT4*-KD switchgrass showed similar molecular weights (*M*_n_ 4570–4890 and *M*_w_ 8770–9700 g mol^−1^) to those of the wild-type switchgrass lignin (*M*_n_ 4490 and *M*_w_ 8930 gmol^−1^) (Supplementary Fig. 8). Also, a comparison of the polydispersity index of the *GAUT4*-KD switchgrass lignin (1.9–2.0) and the wild-type switchgrass lignin (2.0) indicated that molecular weight distribution of the transgenic switchgrass lignin was similar to the wild-type (Supplementary Fig. 8). The measured lignin molecular weights of *GAUT4*-KD switchgrass and the control are comparable with those of *caffeic acid O-methyltransferase* enzyme-suppressed transgenic and control switchgrass^48^.

### Morphology of hydrothermally pretreated *GAUT4*-KD switchgrass

**Fig. 4:**
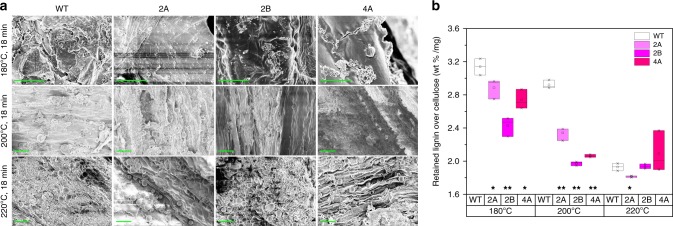
Lignin migration in switchgrass during liquid hot water pretreatment. **a** SEM images of pretreated wild-type (WT) control and *GAUT4*-KD switchgrass (2A, 2B, 4A) at different temperatures. From top to bottom, the rows are images of biomass pretreated at 180 °C, 200 °C, and 220 °C for 17 min, and scale bar is 10 μm. **b** Lignin retained in pretreated biomass. The retained lignin is presented as a ratio of lignin wt% in pretreated biomass over the cellulose weight (mg). *p value <0.05 and **p value <0.001 by Student’s *t* test of biologically duplicate values

Based on the aforementioned association of lower FA content to lignin-hemicellulose disruption, we hypothesized that the disruption of lignin-hemicellulose cross-linkages could facilitate hemicellulose dissolution and lignin migration, leading to lignin droplets during pretreatment. In a previous study, biomass pretreated by hot water and diluted acid revealed a range of droplet morphologies appearing on and within the cell walls of pretreated biomass^49^. The lignin-containing droplets were proposed to form during lignin phase transition by coalescence and migration within and out of the cell wall during pretreatment^49^. Meanwhile, carbohydrates in cell walls could form similar droplets, termed “pseudo-lignin” in dilute acid-pretreated biomass^50^. In addition to the scission of aryl ether linkages in lignin, the cleavage of some labile linkages between lignin and carbohydrates during hydrothermal pretreatment also partially contributed to the disruption of the cell wall matrix and to reduced biomass recalcitrance^15^. To evaluate the effects of *GAUT4* suppression on lignin migration and LCC disruption upon pretreatment, we pretreated the *GAUT4*-KD and the wild-type switchgrass using hot water under different severities by varying the temperature from 180 to 220 °C. SEM images of hydrothermally-pretreated transgenic and control switchgrass were compared to visualize the lignin-containing droplet morphologies (Fig. 4a). In pretreatment at 180 °C, nearly no droplet was visible even at magnifications of 10,000, in either control or transgenic biomass (top row images in Fig. 4a). The surface of pretreated biomass was mostly smooth with the emergence of some “melted” components. By increasing the pretreatment temperature to 200 °C, droplets with different sizes (<3 μm) were readily observed on pretreated biomass at magnifications of 5,000 (middle row images in Fig. 4a). The droplets on biomass increased in size to 3–5 μm when switchgrass was pretreated at 220 °C (bottom row images in Fig. 4a). The results suggested that the formation of droplets was dependent upon the pretreatment severity and the droplet size increased with increased pretreatment temperature. In addition, the droplets on the transgenic lines tended to be more numerous and larger in size when compared with the wild-type control. For instance, the numbers of droplets larger than 1 μm were 15, 14, and 23 per image for the hydrothermally pretreated *GAUT4*-KD lines (at 200 °C), 2A, 2B, and 4A, respectively, while the number of droplets for the wild-type control was 9. These results are consistent with the SEM morphology showing more extensive tissue/cell damage in *GAUT4*-KD lines than in wild-type^10^. However, the transgenic lines showed no favorable droplet formation compared with the control at further increased pretreatment temperature (e.g., 220 °C). This suggests that the mechanisms leading to the easier formation of droplets from the transgenic lines compared with the control was likely compromised at sufficiently elevated pretreatment severities (e.g., at temperatures over a certain threshold). We further quantitated the lignin retained on the pretreated biomass at different temperatures. Since hemicellulose was substantially solubilized during liquid hot water pretreatment, the level of lignin retention in the tissue was presented using the lignin wt% over the cellulose weight which is shown not to be significantly different between the transgenic and wild-type, and for which minimal loss through the pretreatment process is assumed. The results showed that the *GAUT4-*KD lines had lower lignin-retaining quantity compared with the wild-type pretreated at 180 and 200 °C (Fig. 4b). This difference diminished at the higher pretreatment severity of 220 °C, which is consistent with the comparable formation of lignin droplets observed using SEM. This relatively easier formation of droplets implies enhanced disruption of lignin or LCC during pretreatment in the *GAUT4-*KD lines than the control during hot water pretreatment. It could be related to the lower hemicellulose molecular weights and the lower level of LCC cross-linkages, which are favorable for hemicellulose dissociation and lignin migration in the transgenic lines during the pretreatment process.

## Discussion

**Fig. 5:**
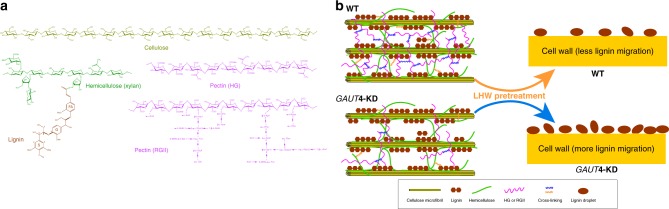
Model of *GAUT4* function in cell wall porosity and biomass recalcitrance. **a** Representative chemical structures of select major wall polymers (cellulose, hemicellulose^51^, lignin^64^, and the pectins HG^11^ and RGII^65^ with modification).(**b** Model depicting reduction of *GAUT4*-synthesized HG in *GAUT4*-KD versus wild-type (WT) biomass and hypothesized consequences on wall porosity and lignin migration during hydrothermal pretreatment. *A hypothesis*: a reduction of HG/RGII in *GAUT4*-KD switchgrass leads to a reduction in cross-linking of polymers between polysaccharides and/or lignin (i.e., reduced FA-based linkage between lignin and hemicellulose (yellow wavy line), and reduced ionic salt bridges and borate diester bonds between HG and RGII (blue wavy line), respectively). This reduced cross-linking results in (1) increased wall porosity and (2) reduced LCC linkages that together lead to easier lignin migration during LHW pretreatment and reduced biomass recalcitrance. FA: ferulate; S: syringyl unit; G: guaiacyl unit; HG: homogalacturonan; RGII: rhamnogalacturonan II; LHW: liquid hot water

Along with the phenotypes of increased biomass yield, reduced recalcitrance, and reduced pectin and pectin biosynthesis^10^, we show here that KD expression of the *GAUT4* gene in switchgrass also affects hemicellulose molecular weights, lignin structure, and LCC cross-linkages in switchgrass, which likely contributes to the reduced recalcitrance phenotype. Although the chemical compositions, for example, the contents of neutral sugars and lignin, were similar between the transgenic and control plants, the *GAUT4*-KD lines had reduced hemicellulose molecular weights revealed by size-exclusion chromatographic analysis and reduced FA content, as well as fewer LCC linkages supported by reduced arabinan/arabinose and xylan/xylose residual in lignin-enriched residues revealed by 2D HSQC NMR spectra. Associated with these structural differences in hemicellulose and lignin, the *GAUT4*-KD lines demonstrated a lower lignin-retaining capability and an easier migration and coalescence of lignin-like droplets after hydrothermal pretreatment. Taking together, the recalcitrance reduction in *GAUT4*-KD switchgrass lines and the increased accessibility to cellulase are likely associated with two factors (Fig. 5): (1) reduced pectic homogalacturonan and rhamnogalacturonan II contents and cross-linking leading to higher cell wall porosity^10^; and (2) reduced LCC linkages and hemicellulose molecular weight which are favorable to lignin (or pseudo-lignin) migration out of the cell wall during pretreatment.

Within the complex cell wall matrix of polysaccharides, phenolics, and proteins in grasses, FA plays an important role in both cross-linking hemicellulosic polysaccharides and in cell wall lignification^51^. Some arabinogalactan proteins in *Arabidopsis thaliana* have been shown to be covalently cross-linked with pectic and hemicellulosic polysaccharides including homogalacturonan and rhamnogalacturonan I^52^. Therefore, the reduction of homogalacturonan and rhamnogalacturonan II in the *GAUT4*-KD lines might affect the association of pectin and xylan inter-polymer cross-linking and lead to a reduced molecular weight of the isolated hemicellulose. This would contribute to the hemicellulose dissolution and, at least, increased cell wall porosity during the hot water pretreatment. On the other hand, increasing evidence indicates that pectin structures are associated closely with lignin. Pectin such as homogalacturonan and rhamnogalacturonan II consists of poly-α-(1→4)-galacturonic acid backbones that are free to form both ester and ether linkages with lignin. Characteristic pectic β-1,4-linked d-galactan and α-1,5-linked l-arabinan have been detected in wood lignin, residual lignin from Kraft pulping, and middle lamella-enriched fractions from pine lignin^13^. Analyses of an LCC fraction from birchwood meal indicated that most of the hemicellulose in the LCC fraction from the middle lamella, especially the xylan, was associated with lignin through pectin. The results supported the proposal that a carboxyl group of GalA may be involved in a benzyl ester bond between lignin and pectin^53^. Lawoko et al.^54^ showed that treatment of LCCs from the middle lamella of softwood with pectinase led to a shift to a higher molar mass distribution of lignin, suggesting that pectin has a role in regulating the shape of lignin in the wall^54^. Treatment of birch LCC with a purified endopectate lyase resulted in significantly increased lignin content in the residuals^55^. Wi et al.^14^ found that the inhomogeneity in lignin distribution in the middle lamella region of alfalfa secondary xylem fibers, where much of the pectin is located and where lignification is initiated, may be related to the irregular pattern of pectin distribution. A biomimetic study of lignin-pectin particles synthesis has revealed that the content of pectins strongly associated with the formation of bonding system (i.e., condensed bonds and β-*O*-4 ether bond) in lignification, although the fundamental mechanisms underlying this influence of pectin remain unclear^56^. These results suggest that pectins may cross-link portions of lignin to hemicellulose and affect the lignification process in the herbaceous species.

It has been suggested that calcium in cambial tissue is important for the lignification of cell walls and that pectin may serve a regulatory role due to the high concentration of calcium in homogalacturonan:homogalacturonan salt bridges^13^. When the cambium resumes cell division and expansion in the spring, calcium bridges in acidic pectin in the middle lamella have to be degraded, making calcium available ^57^. Calcium is suggested to be involved in lignin polymerization in developing xylem within the cell wall. Important key enzymes during lignification such as peroxidases are known to bind pectin in their calcium-induced structure. An irregular distribution of peroxidase and H_2_O_2_ further support the hypothesis that the inhomogeneity in pectin architecture may be associated with the irregular pattern of the lignin deposition^14^. Thus, the biosynthesis of pectin and lignin may be connected through the involvement of calcium ions and peroxidase localization. In our prior study, we showed that down-regulation of *GAUT4* decreases homogalacturonan-Ca^2+^-salt bridges in the cell wall^10^. Therefore, a variation of lignification in the form of reduced cross-linking with carbohydrates may be one of the responses to KD expression of *GAUT4* in switchgrass. However, the direct connection between pectin biosynthesis and LCC structure, particularly the reduced level of FA in lignin, remains to be determined.

The results provide insight into structural changes of cell wall components associated with suppression of *GAUT4* gene expression in switchgrass and their significance to reduced cell wall recalcitrance. More broadly, the results show that modified expression of even a single cell wall biosynthetic gene in plants can result in multiple interrelated changes in plant cell wall structure, cross-linking, and recalcitrance to pretreatment and deconstruction strategies and highlight the importance of studying the multiple chemical and physical wall parameters that may be affected by each unique cell wall modification.

## Methods

### Generation and growth of transgenic switchgrass

A lowland-type switchgrass cultivar, Alamo (*Panicum virgatum* L.), was genetically modified by KD expression of the *GAUT4* gene as described previously^10^. In brief, Switchgrass contig AP13CTG20100 was identified and was used to design primers for cloning. The RNA interference (RNAi) cassettes were constructed by isolating a 443-bp *GAUT4* fragment from switchgrass (*P. virgatum* L.) followed by cloning into pCR8 entry vector for sequence confirmation and recombining into pANIC12A. Embryogenic callus derived from immature inflorescences of SA7 was transformed with the expression vector construct through biolistic transformation. SA7 was developed for improved tissue culture response and selected from a cross of two regenerable “Alamo” genotypes, ST1 and Alamo2. Transgenic lines were confirmed by PCR with vector primers flanking the *GAUT4* RNAi hairpin inserts. The *GAUT4*-KD transgenic switchgrass lines (2A, 2B, and 4A) and the wild-type control were grown in a greenhouse (University of Georgia, GA, USA). Switchgrass whole tillers at the R1 stage from 3-month-old plants were harvested and air-dried at room temperature for 3–4 weeks (depending on the season) and subsequently milled and screened to 0.85 mm of particle size using a Wiley Mini-Mill (model number: 3383L10, Thomas Scientific). All samples were biological duplicates and all quantitative analysis included at least technical duplicates (only biological duplicate were quantified for NMR data).

### SEM analysis

In order to visualize the lignin migration and coalescence, the air-dried and milled switchgrass samples were subjected to hot water pretreatment using differing degrees of severity. To minimize effects of inconsistent ramping times, all samples including *GAUT4-*KD (2A, 2B, and 4A) and wild-type control switchgrass were pretreated in one batch reactor. Each biomass sample was loaded into a sealed glass vial with 1:50 of the solid to liquid ratio, and the vials were placed in a Parr reactor for the pretreatment. The pretreatment was conducted at different temperatures (180, 200, and 220 °C) for 17 min as previously described^58^. The ramping times were 24, 28, and 35 min to reach 180, 200, and 220 °C, respectively, and the cooling time was ~2 min to room temperature in icy water. The images of lignin-like droplets in pretreated switchgrass were viewed using a Leo 1525 FE-SEM. The pretreated samples were air-dried and mounted on aluminum stubs using carbon tape and samples were then sputter-coated with 10–12 nm Au using an SPI-Module Sputter Coater for 50 s. Imaging was performed at 3 kV and at various magnifications.

### Lignin content analysis

The lignin content in nonpretreated and liquid hot water-pretreated switchgrass was measured using the acetyl bromide soluble lignin (ABSL) assay^59,60^. In brief, pretreated switchgrass was collected via filtration using filter paper and washed with deionized (DI) water and ethanol to remove surface-deposited lignin. About 30 mg of air-dried untreated and pretreated samples were milled in a Retsch Vibration Mill MM 200 at 15 Hz for 30 min. The milled samples were vacuum dried at 40 °C for 48 h before the lignin content was quantified using the ABSL assay. The lignin retained in the pretreated switchgrass was presented as the ratio of measured lignin weight percentage over cellulose mass (mg).

### Isolation of hemicellulose-enriched fraction

The hemicellulose-enriched fraction was isolated from switchgrass biomass as described in Kumar et al.^61^ (Supplementary Fig. 9). The extractives-free samples were delignified using peracetic acid with 5.00 g loading per g biomass. The solution consistency was adjusted to 5% with DI water and holopulping was conducted at room temperature for 24 h with magnetic stirring. The solid residue, designated as holocellulose, was washed with excessive DI water (18.0MΩ) and air-dried at room temperature for 24 h. The air-dried holocellulose (100 mg) was consecutively extracted at 25°C with 17.5% (w/v) NaOH solution (5.00 mL) for 2 h, followed by 8.75% NaOH solution (10.00 mL) for an additional 2 h. The alkaline slurry was filtered and rinsed with 5 mL of 1% acetic acid leading to a liquid fraction and a solid residue. The liquid fraction, rich in hemicellulose, was adjusted to pH 6–7 with anhydrous acetic acid. The hemicellulose-enriched fraction, designated alkaline hemicellulose, was then precipitated by adding three volumes of 100% ethanol to the liquid fraction and the hemicellulose was obtained by centrifugation at 8000 rpm (267π rad s^−1^) for 5 min and freeze-dried for 24 h.

### Isolation of CEL

CEL was isolated as illustrated in Supplementary Fig. 9. Extractives-free switchgrass biomass was prepared from the Wiley-milled samples by Soxhlet extraction with an ethanol:toluene mixture (1:2, v/v) for 24 h followed by acetone extraction for an additional 12 h. About 1 g of the extractives-free sample was loaded into a ZrO_2_ grinding jar (internal volume: 50 mL) with 10 ZrO_2_ balls in a Retsch Ball Mill PM 100. The biomass was ball milled at 580 rpm at a frequency of 5 min with 5 min pauses in-between for a total time of 1.5 h. The ball-milled cell wall powder was then subjected to enzymatic hydrolysis with a mixture of Cellic^®^ CTec2 and HTec2 (Gift from Novozymes) in the sodium acetate buffer solution (pH 4.8) at 50 °C under continuous agitation at 200 rpm for 48 h. The residual solids were isolated by centrifugation and hydrolyzed once more with freshly added enzyme mixture. The residual solids, called lignin-enriched residue, obtained after two-step hydrolysis were washed with DI water, centrifuged, and freeze-dried. The lignin-enriched residue was extracted with dioxane-water (96% v/v, 10.0 mL g^−1^ biomass) for 24 h. The extracted mixture was centrifuged and the supernatant was collected. Dioxane extraction was repeated once with fresh dioxane water. Dioxane and water were evaporated using rotary evaporator at 45 °C and the residuals were freeze-dried. The obtained cellulolytic enzyme lignin samples, designated as CEL, were used for the further analysis.

### Analysis of glycosyl residue composition

Glycosyl residue composition of the isolated hemicellulose and lignin was determined by gas chromatography-mass spectrometry (GC-MS) of trimethylsilyl (TMS) derivatives^10^. In brief, approximately 500 μg of isolated hemicellulose and lignin fractions from switchgrass wild-type and the *GAUT4*-KD lines (as described above) were hydrolyzed for 18 h at 80 °C in 1 M methanolic-HCl. The released glycosyl residues were derivatized with 200 μL TriSil reagent at 80 °C for 20 min. The resulting TMS methyl glycosides were analyzed by GC-MS using an Agilent 7890A GC interfaced to a 5975C MSD (mass selective detector, electron impact ionization mode). Separation was performed on a Supelco EC-1 fused silica capillary column (30 m × 0.25 mm ID) using helium as carrier gas.

### GPC analysis

The weight-average molecular weight (*M*_w_) and number-average molecular weight (*M*_n_) of lignin were measured by GPC after acetylation as described in a previous study^48^. Briefly, the derivatization of lignin was conducted on a basis of ~3 mg lignin in 1 mL of 1:1 v/v pyridine/acetic anhydride in the dark at room temperature for 24 h under magnetic stirring. The solvent/reagents were removed by co-evaporation at 45 °C with ethanol, several times, using a rotatory evaporator. The acetylated lignin was dissolved in tetrahydrofuran and the solution was filtered through a 0.45μm membrane filter before GPC analysis. Size-exclusion separation was performed on an Agilent 1200 HPLC system (Agilent Technologies, Inc., Santa Clara, CA, US) equipped with Waters Styragel columns (HR1, HR2, and HR6; Waters Corporation, Milford, MA, USA) and ultraviolet detector (wavelength: 270 nm). Tetrahydrofuran was used as the mobile phase at a flow rate of 1.0 mL min^−1^. Polystyrene standards were used for establishing the calibration curve.

The molecular weights of hemicellulose were measured using GPC equipped with Waters Ultrahydrogel columns (120, 250, 500; Waters Corporation, Milford, MA, USA) and refractive index detector . Aqueous buffer was used as the mobile phase at a flow rate of 0.5 mL min^−1^. Pullulan standards were used for establishing the calibration curve. The freeze-dried hemicellulose samples were dissolved in 0.05 M sodium hydroxide per 0.1 M sodium acetate (pH 12.0) mobile phase (~1.0 mg mL^−1^) directly and filtered through a 0.45 µm filter before GPC analysis.

### NMR spectroscopic analysis

NMR spectra of isolated lignin samples were acquired in a Bruker Avance/DMX 400 MHz spectrometer and spectral processing used Bruker’s Topspin 3.5 (Mac) software. A standard Bruker HSQC pulse sequence (hsqcetgp) was used on a BBFO probe with the following acquisition parameters: spectra width 10 ppm in F2 (^1^H) dimension with 2048 time of domain (acquisition time 256.1 ms), 210 ppm in F1 (^13^C) dimension with 256 time of domain (acquisition time 6.1 ms), a 1.5-s delay, a ^1^*J*_C–H_ of 145 Hz, and 32 scans. The central dimethyl sulfoxide solvent peak (δ_C_/δ_H_ at 39.5/2.49) was used for chemical shift calibration. Relative abundance of lignin compositional subunits and inter-unit linkage were estimated semi-quantitatively using volume integration of contours in HSQC spectra^44,62^. For monolignol compositions of S, G, H, *p*-coumarate, FA, and tricin quantitation, the S_2/6_, G_2_, H_2/6_, *p*CA_2/6_, FA_2_ and T_2'/6'_ were integrated. The Cα signals were used for contour integration for inter-unit linkages estimation.

For quantitative ^31^P NMR, CEL was phosphitylated with 2-chloro-4,4,5,5-tetramethyl-1,3,2-dioxaphospholane in a solvent of pyridine/CDCl_3_ (1.6/1.0, v/v) according to the published method in Granata and Argyropoulos^63^. In detail, 20.0 mg of CEL was weighed into a 4-mL vial sealed with polytetrafluoroethylene cap. A prepared stock solution of pyridine/deuterated chloroform (500 μL) including 1 mg/mL Cr_(acac)3_ and 4 mg mL^−1^ internal standard (endo *N*-hydroxy-5-norbene-2,3-dicarboxylic acid imide) was added to dissolve lignin. The phosphitylation was performed by adding 50 µL of the phosphitylating reagent, 2-chloro-4,4,5,5-tetramethyl-1,3,2-dioxaphospholane. Quantitative ^31^P NMR spectra were acquired on a Bruker Avance 400 MHz spectrometer equipped with a BBO (broadband observe) probe using an inverse-gated decoupling pulse sequence (Waltz-16), 90° pulse, 25-spulse delay with 64 scans. All chemical shifts reported are relative to the product of 2-chloro-4,4,5,5-tetramethyl-1,3,2-dioxaphospholane with water, which has been observed to give a sharp signal in pyridine/CDCl_3_ at 132.2 ppm. The contents of OHs were quantitated on the basis of the amount of added internal standard.

### Statistical analysis

All statistical analyses were made with Microsoft Excel 2010. The *t* test was applied with equal or unequal variances based on the results of an F test for each comparison. A two-tail *p* value <0.05 indicates changes between compared groups were significant at 95% confidence level.

## Supplementary information

Supplementary Information

Supplementary Data 1

Description of Supplementary Files

## Data Availability

All data generated or analyzed during this study are included in this published article and its supplementary information files. Source data for figures and supplementary figures are available in Supplementary Data 1.
