# Supplementary material for: Downregulation of pectin biosynthesis gene GAUT4 leads to reduced ferulate and lignin-carbohydrate cross-linking in switchgrass
Source: Commun Biol. 2019 Jan 17;2:22. doi: 10.1038/s42003-018-0265-6 (PMC6336719; doi:10.1038/s42003-018-0265-6)
Supplement: Supplementary file 1 — Supplementary Information [file 42003_2018_265_MOESM1_ESM.docx]

**Supplementary Figure 1**

2D HSQC NMR spectra of hemicellulose isolated from *GAUT4*-KD transgenic switchgrass (2A, 2B, 4A) and wild-type (WT) control. The contours are colored to match the names according to the assignment in previous publications.^1-3^

**Supplementary Figure 2**

^13^C NMR spectra of hemicellulose isolated from *GAUT4*-KD transgenic switchgrass (2A, 2B, 4A) and wild-type (WT) control. The peak assignments were made according to the literature. ^1,2^

**Supplementary Figure 3**

The mass yield of isolated cellulolytic enzyme lignin (CEL) from extractives-free switchgrass on an oven-dry basis. The CEL obtained represents 20-24 *wt*% Klason lignin content of the biomass. Mass yields are average values of duplicate measurements including standard deviation.

**Supplementary Figure 4**

The glycosyl residue composition of the CEL fractions obtained by the TMS method. * *p*-value <0.05 and ** *p*-value <0.001 by Student’s *t*-test of biologically duplicate values.

**Supplementary Figure 5**

Aliphatic regions from 2D HSQC NMR spectra of lignin isolated from transgenic switchgrass (*GAUT4*-KD) and control (WT). The contours are colored to match the structures. PhGlc: phenyl glycoside linkages.

**Supplementary Figure 6**

Relative peak intensities of anomeric carbon from sugar units (Ara: arabinan; Xyl: xylan) to total lignin aromatic (Ar) subunits.

**Supplementary Figure 7**

^31^P NMR spectra of phosphitylated lignin from *GAUT4*-KD transgenic switchgrass (2A, 2B, 4A) and wild-type (WT) control.

**Supplementary Figure 8**

Lignin molecular weight. Insert is lignin polydispersity index (PDI). Students’ *t-test* showed no significant difference between the *GAUT4*-KD lines and WT control at a 95% confidence level. *M_n_*: number-average molecular weight; *M_w_*: weight-average molecular weight.

**Supplementary Figure 9**

Overview of the isolation and analyses of hemicellulose and cellulolytic enzyme lignin (CEL) from switchgrass. PAA: peracetic acid; HOAc: acetic acid; NaOH: sodium hydroxide.

1 Nie, X.-N., Liu, J., She, D., Sun, R.-C. & Xu, F. Physicochemical and Structural Characterization of Hemicelluloses Isolated by Different Alcohols from Rice Straw. *BioResources* **8**, 3817-3832 (2013).

2 Peng, P., Peng, F., Bian, J., Xu, F. & Sun, R. Studies on the starch and hemicelluloses fractionated by graded ethanol precipitation from bamboo Phyllostachys bambusoides f. shouzhu Yi. *J. Agric. Food. Chem.* **59**, 2680-2688 (2011).

3 Kim, H. & Ralph, J. A gel-state 2D-NMR method for plant cell wall profiling and analysis: a model study with the amorphous cellulose and xylan from ball-milled cotton linters. *Rsc Advances* **4**, 7549-7560 (2014).
