# Supplementary material for: Downregulation of pectin biosynthesis gene GAUT4 leads to reduced ferulate and lignin-carbohydrate cross-linking in switchgrass
Source: Commun Biol. 2019 Jan 17;2:22. doi: 10.1038/s42003-018-0265-6 (PMC6336719; doi:10.1038/s42003-018-0265-6)
Supplement: Supplementary file 3 — Description of Supplementary Files [file 42003_2018_265_MOESM3_ESM.docx]

**Description of Supplementary Data 1**

Supplementary Data 1 provides the original data for Fig. 1 The molecular weights and glycosyl residue composition of the hemicellulose fractions from switchgrass; Fig. 2 Relative abundance of lignin aromatic units in the GAUT4-KD and control switchgrass; Fig. 3 Lignin hydroxyl groups analyzed using ^31^P NMR after phosphitylation: Fig. 4b Lignin retained in pretreated biomass; Supplementary Fig. 4 The glycosyl residue composition of the CEL fractions obtained by the TMS method; Supplementary Fig. 6 Relative peak intensities of anomeric carbon from sugar units to total lignin aromatic subunits; Supplementary Fig. 8 Lignin molecular weight and polydispersity index.
